# Contribution of comorbid pathologies to amyotrophic lateral sclerosis with cognitive or behavioral abnormalities

**DOI:** 10.1186/s12883-025-04556-z

**Published:** 2025-12-03

**Authors:** Hideyuki Moriyoshi, Akio Akagi, Yuichi Riku, Jun Sone, Hiroaki Miyahara, Mari Yoshida, Masahisa Katsuno, Yasushi Iwasaki

**Affiliations:** 1 Department of Neuropathology, Institute for Medical Science of Aging, Aichi Medical University, 1-1 Yazakokarimata, Nagakute, Aichi 480-1195 Japan; 2 Department of Neurology, Nagoya University Graduate School of Medicine, 65 Tsurumai-cho, Showa-ku, Nagoya, Aichi 466-8550 Japan

**Keywords:** Amyotrophic lateral sclerosis, Alzheimer’s disease, Argyrophilic grain disease, Senile dementia of the neurofibrillary tangle type, Primary age-related tauopathy, Frontotemporal lobar degeneration

## Abstract

**Background:**

Amyotrophic lateral sclerosis (ALS) often presents with cognitive or behavioral abnormalities. The cortical involvement of TAR DNA-binding protein-43 (TDP-43) pathology is considered a major cause of these abnormalities. However, the contribution of underlying comorbid pathologies remains unclear.

**Methods:**

We investigated the clinicopathological characteristics of 29 autopsy cases of ALS with cognitive or behavioral abnormalities and evaluated the association between clinical symptoms and comorbid pathologies such as Alzheimer’s disease (AD), argyrophilic grain disease (AGD), dementia with Lewy bodies (DLB), and primary age-related tauopathy (PART), as well as the presence of cortical TDP-43 pathology.

**Results:**

Of the 29 patients, 17 exhibited comorbid pathologies (AD, AGD, or PART), which may contribute to cognitive or behavioral abnormalities. None of the cases met the pathological criteria for DLB. The group with comorbid pathologies was significantly older, but clinical symptoms did not differ between the groups. Behavioral abnormalities and memory impairment were frequently observed in both groups. All six subjects without cortical TDP-43 pathology had comorbid pathologies, which had a notable effect on cognitive or behavioral abnormalities. Hippocampal sclerosis and memory impairment were observed in ALS cases without comorbid pathologies.

**Conclusion:**

A high frequency of comorbid pathologies is observed in elderly patients with ALS presenting with cognitive or behavioral abnormalities. There are cases of ALS in which comorbid pathologies such as AD, AGD, and PART may contribute to cognitive or behavioral abnormalities, even in the absence of cortical TDP-43 pathology. Hippocampal sclerosis of ALS may contribute to memory impairment independently of comorbid pathologies.

## Background

Amyotrophic lateral sclerosis (ALS) is a progressive neurodegenerative disorder that affects the upper and lower motor neurons, leading to generalized muscle weakness and respiratory impairment [1]. Furthermore, cognitive or behavioral abnormalities occur in 30–50% of patients with ALS, and approximately 15% meet the diagnostic criteria for frontotemporal dementia (FTD) [1]. The key pathological feature of ALS is the abnormal accumulation of TAR DNA-binding protein 43 (TDP-43) in the affected neurons [2, 3]. TDP-43 pathology often extends beyond the motor neuron system into the frontotemporal lobe, overlapping with the pathological spectrum of frontotemporal lobar degeneration (FTLD) [4, 5]. Additionally, TDP-43 pathology in the frontotemporal lobes is a major cause of cognitive or behavioral abnormalities in ALS. However, comorbid pathologies such as Alzheimer’s disease (AD) also contribute to cognitive decline [6, 7]. The contribution of the comorbid pathology to ALS with cognitive or behavioral abnormalities remains unclear because postmortem examination is necessary for accurate diagnosis. Therefore, we aimed to clarify the contribution of comorbid pathologies to ALS with cognitive or behavioral abnormalities.

## Methods

### Subjects

We retrospectively screened the clinicopathological data of 153 consecutive autopsy cases of ALS from 2010 to 2023 at the Institute for Medical Science of Aging, Aichi Medical University. Although a structured longitudinal clinical follow-up was not performed, all patients were regularly followed by neurologists during routine clinical practice. The data used in this study were obtained from these clinical records. Cognitive or behavioral abnormalities in all patients were reported by the neurologist based on clinical examination and caregiver complaint. Among the 153 patients, 31 with any cognitive or behavioral abnormalities described in the clinical records were included in this study. The remaining 122 patients were excluded either for having normal cognitive function or no documentation of cognitive or behavioral abnormality. Two patients were excluded: One was excluded owing to insufficient clinical data and limited tissue availability, and the other was excluded given that the described behavioral abnormality was deemed to reflect innate self-directed personality. Therefore, 29 patients were finally analyzed. All patients met the criteria for “possible” or more advanced categories according to the revised El Escorial criteria for ALS diagnosis [8]. Written informed consent was obtained from patients’ relatives before the autopsy. The Research Ethics Committee of Aichi Medical University approved all investigations, which were conducted in accordance with all provisions of the Declaration of Helsinki.

### Clinical assessments

We retrospectively assessed clinical information from medical records, including age at onset, age at death, sex, total disease duration, onset time of cognitive or behavioral abnormalities, and initial motor symptoms. Because standardized ALS-specific neuropsychological assessments were unavailable for most patients, cognitive or behavioral abnormalities were classified according to the clinical descriptions. The cognitive abnormalities were categorized into the following domains: language dysfunction, executive dysfunction, and memory disturbance [9]. Language dysfunction included cases clinically diagnosed with progressive non-fluent aphasia or semantic dementia. Executive dysfunction was defined as impairment in executive functions, such as planning, judgment, problem-solving, cognitive flexibility, which significantly interfered with work or daily life activities. Memory impairment was defined as disturbance of short-term memory or episodic memory. Behavioral abnormalities include disinhibition, irritability, personality changes, apathy, altered food preferences, and perseverative behaviors. The diagnosis of ALS-FTD was based on proposed criteria [9].

### Tissue Preparation

The left hemisphere of the brain and the spinal cord were fixed in 20% neutral-buffered formalin for at least 2 weeks. The left cerebral hemisphere was sectioned coronally at a thickness of 8 mm, the brainstem and spinal cord were sectioned transversely, and the cerebellum was sectioned sagittally at a thickness of 5 mm. Subsequently, the regions of interest were trimmed and embedded in paraffin. Sections of 9 μm thickness were prepared for hematoxylin-eosin (HE), Klüver-Barrera, and Gallyas-Braak staining. Sections of 4.5 μm thickness were prepared for immunohistochemical analysis. The primary antibodies used for immunohistochemistry were phosphorylated tau (1:4000, mouse monoclonal, clone AT8; Thermo Scientific, Rockford, IL, USA), amyloid-β (1:1000, mouse monoclonal, clone 12B2; IBL, Gunma, Japan), phosphorylated α-synuclein (1:6000, mouse monoclonal, pSyn#64; Wako Pure Chemical Industries, Osaka, Japan), and phosphorylated TDP-43 (1:4000, rabbit polyclonal; Cosmo Bio, Tokyo, Japan).

### Neuropathological evaluation

**Fig. 1:**
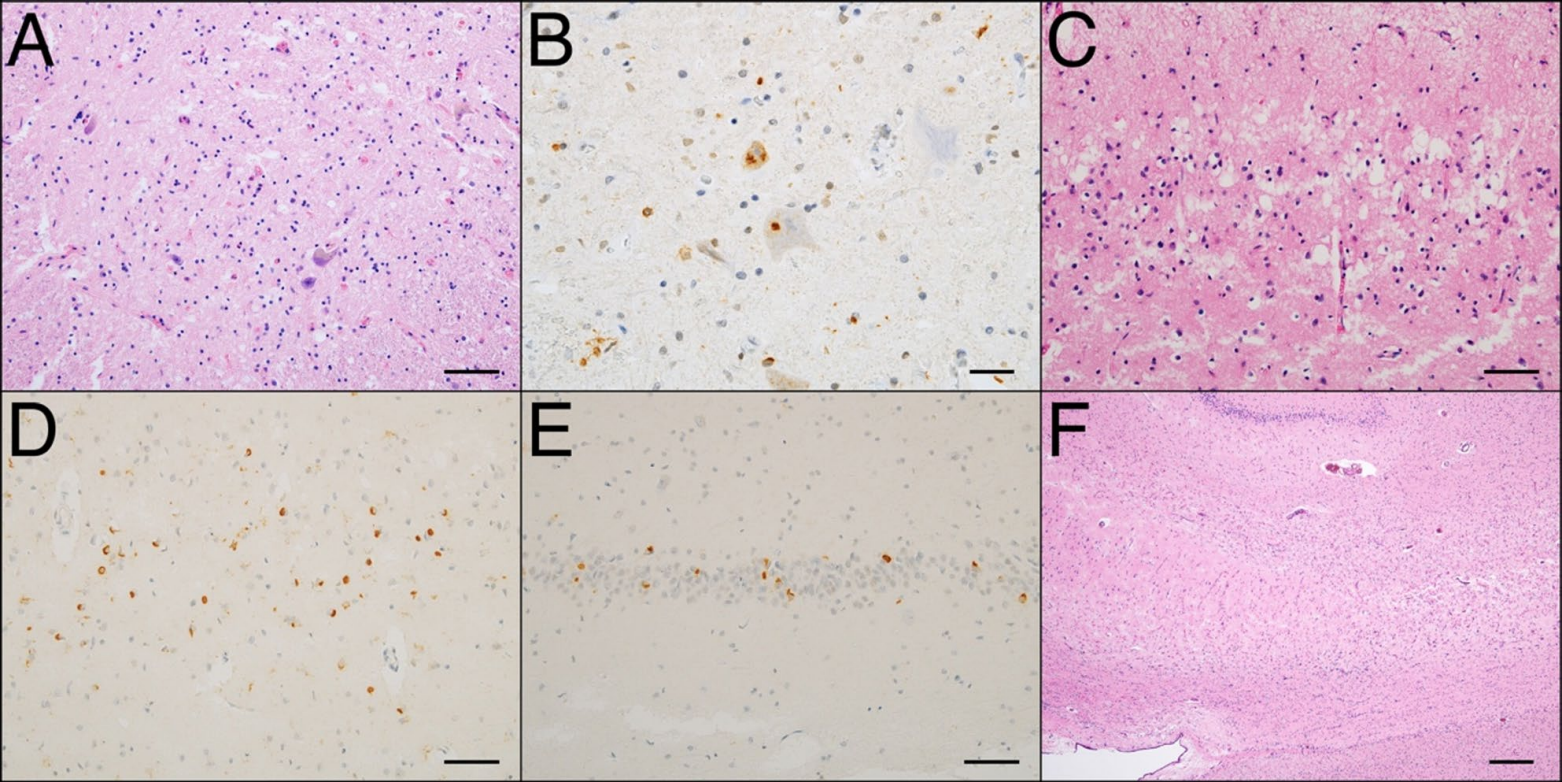
Microscopic appearance of amyotrophic lateral sclerosis (ALS) type 2. (**A**, **C**, **F**) Hematoxylin and eosin staining. (**B**, **D**–**E**) Immunohistochemistry for phosphorylated TAR DNA-binding protein 43 (pTDP-43). (**A**) Severe neuronal loss and astrogliosis of the anterior horn of the cervical spinal cord. (**B**) Abnormal cytoplasmic accumulation of pTDP-43 in the anterior horn of the cervical spinal cord. (**C**) Microvacuolization of superior layer of temporal lobe. (**D**) Abnormal cytoplasmic accumulation of pTDP-43 of temporal cortex. (**E**) Abnormal cytoplasmic accumulation of pTDP-43 in the hippocampal dentate gyrus. (**F**) Hippocampal sclerosis characterized by severe neuronal loss and astrogliosis in the subiculum. Bar = 200 μm (**F**), 50 μm (**A**, **C**–**E**), 20 μm (**B**)

The pathological diagnosis of ALS was established based on neuronal loss and astrogliosis in the anterior horn of the spinal cord and the precentral gyrus in association with TDP-43 pathology (Fig. 1A, B). We classified the cases into two subtypes (ALS types 1 and 2) based on their distribution pattern [4]. ALS type 1 is primarily localized to the motor neuron system, although a slight spread beyond the system occurs. ALS type 2 is characterized by the significant involvement of the frontotemporal cortex accompanied by cortical neuronal loss, astrogliosis, and microvacuolization (Fig. 1C–E). Cortical TDP-43 pathology was classified into three subtypes according to the FTLD-TDP classification [10]. In addition, TDP-43 pathology was classified into five hierarchical stages based on previous reports [11, 12]. Hippocampal sclerosis (HS) in ALS was assessed based on neuronal loss and gliosis in the subiculum (Fig. 1F) [13]. Comorbid pathology was assessed for AD, primary age-related tauopathy (PART), Lewy body disease (LBD), and argyrophilic grain disease (AGD). Neurofibrillary tangles (NFTs) were evaluated using Braak’s stage [14]. Amyloid-β deposition was evaluated in five stages according to the Thal phase [15]. The density of the senile plaque was classified into four grades according to the consortium to establish a registry for Alzheimer’s disease (CERAD) [16]. The neuropathological diagnosis of AD was made for at least intermediate AD neuropathologic change, according to the National Institute on Aging-Alzheimer’s Association criteria [17]. PART was assessed according to the proposed criteria by using Braak’s NFT stage. NFTs at Braak Stage III or higher were considered to contribute to cognitive or behavioral abnormalities corresponding to senile dementia of neurofibrillary tangle-type (SD-NFT) [18–20]. LBD was screened by HE staining to identify Lewy bodies in the dorsal motor nucleus of the vagus, locus coeruleus, substantia nigra, and amygdala. α-Synuclein immunostaining was conducted in the dorsal motor nucleus and amygdala, and if positive, further staining of the brainstem, limbic regions, and cortex was performed according to the guidelines [21]. AGD were categorized into three grades using Gallyas-Braak staining [22].

### Statistical analyses

Statistical analyses were performed using R software (version 4.4.1; R Foundation for Statistical Computing, Vienna, Austria). The clinical and pathological findings were compared between ALS patients with and without comorbid pathologies using the Mann–Whitney U and Fisher’s exact tests for quantitative and qualitative variables, respectively. The significance level was set at 0.05 for comparisons between the two groups. All statistical analyses were two-sided.

## Results

**Fig. 2:**
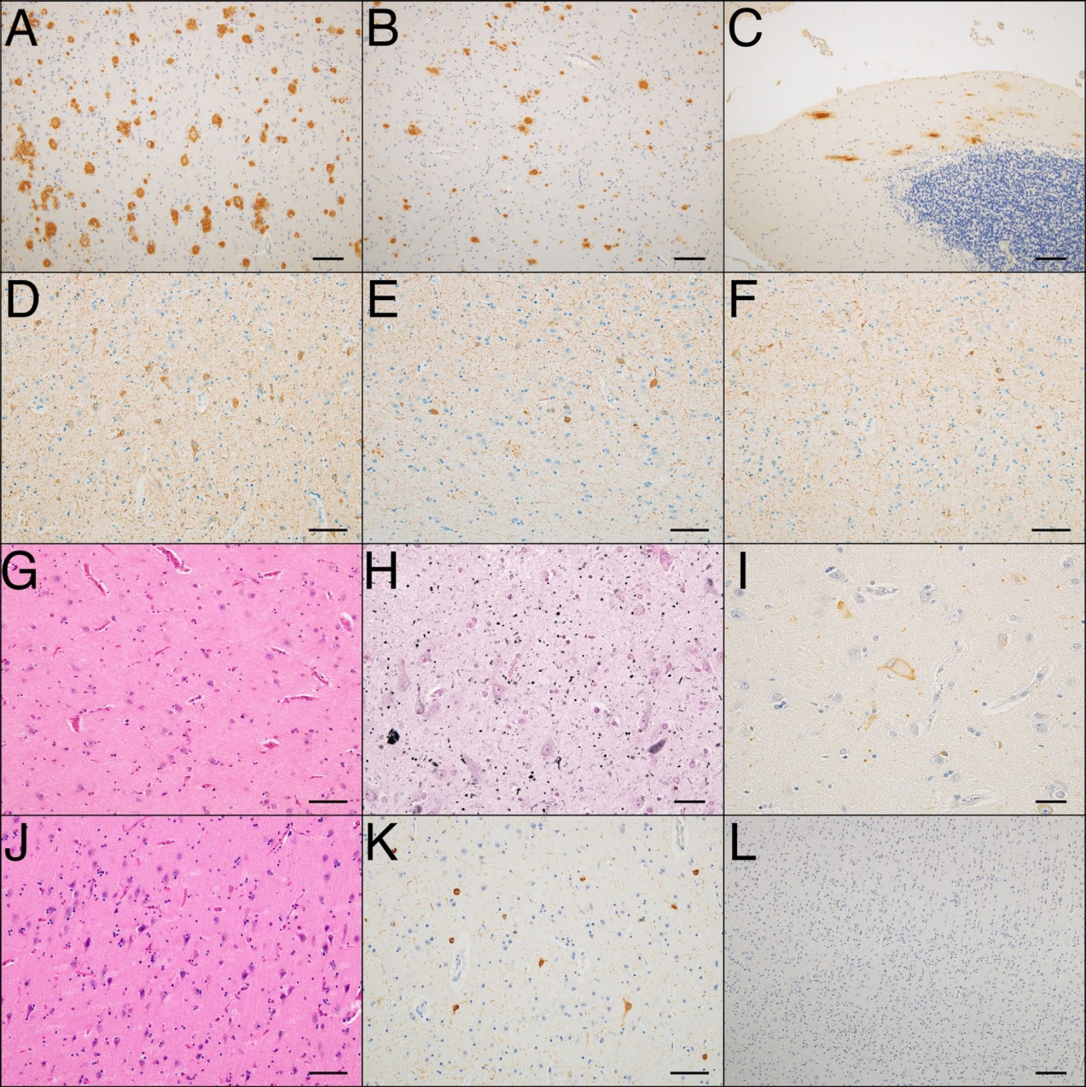
Microscopic appearance of comorbid pathology. (**A**–**F**) Case 1 diagnosed with Alzheimer’s disease. (**A**–**C**) Immunohistochemistry (IHC) for amyloid β (Aβ); (**A**) frontal cortex, (**B**) putamen, and (**C**) cerebellum. Widespread Aβ deposition is observed throughout the cerebrum, consistent with Thal phase 5. (**D**–**F**) IHC for phosphorylated tau (pTau); (**D**) Entorhinal region, (**E**) occipitotemporal gyrus, and (**F**) superior temporal gyrus show numerous neurofibrillary tangles (NFTs) and neuropil threads consistent with Braak NFT stage V. (G–I) Case 4 diagnosed with argyrophilic grain disease. (**G**) Hematoxylin and eosin (HE) staining of the amygdala shows moderate neuronal loss and gliosis. (**H**) Gallyas-Braak staining in the subiculum reveals numerous argyrophilic grains. (**I**) IHC for pTau of the amygdala demonstrates ballooned neurons. (**J**–**L**) Case 5 diagnosed with primary age-related tauopathy at Braak NFT stage III. (**J**) HE staining shows severe astrogliosis in the entorhinal cortex. (**K**) IHC for pTau shows numerous NFTs in the occipitotemporal cortex, with a density greater than that observed in the AD case. (**L**) IHC of the occipital cortex reveals no Aβ deposition. Bar = 100 μm (**A**–**C**, **L**), 50 μm (**D**–**G**, **J**–**K**), 20 μm (**H**–**I**)

Table 1 compares the clinical and pathological features of ALS with and without comorbid pathologies. The study sample consisted of relatively older individuals, with a median age at death of 74 years, and included a slightly higher proportion of males. The median total disease duration was 40 months. In over 50% of the cases, cognitive or behavioral abnormalities appeared before the onset of motor symptoms. Notably, most initial motor symptoms began in the bulbar region. Behavioral abnormalities were the most common, observed in approximately 70% of the cases, followed by memory impairment in approximately 40%. Among the behavioral abnormalities, irritability was the most frequent one. Language impairment and executive dysfunction were observed in only a few cases. Three patients were diagnosed progressive nonfluent aphasia. Only two patients met the diagnostic criteria for behavioral variant FTD. Five patients met the diagnostic criteria for ALS-FTD. Approximately 80% of the cases were classified as ALS type 2. In most of the ALS type 2 patients, the TDP-43 pathology was FTLD type B. HS was observed in approximately 50% of the cases. Of the 29 cases, 17 had a comorbid pathology, such as AD, AGD, and PART, with Braak’s NFT stage Ⅲ. Four cases had a high-likelihood AD pathology, and five had an intermediate-likelihood AD pathology, totaling nine cases (Fig. 2A–F). AGD was diagnosed in four cases: two at stage I, and one each at stages II and III (Fig. 2G–I). Four cases of PART were classified as Braak’s NFT stage Ⅲ (Fig. 2J–L). None of the cases had α-synuclein pathology consistent with dementia with Lewy bodies, although one case showed a small number of α-synuclein positive structures in the medulla oblongata. The age at onset tended to be higher in the group with comorbid pathologies, although the difference did not reach statistical significance (*p* = 0.056). The age at death was significantly higher in the group with comorbid pathologies (*p* = 0.048). The frequency of ALS type 2 was significantly higher in the group without comorbid pathology (*p* = 0.028). No significant differences were observed between groups in sex distribution, disease duration, timing of dementia onset, initial motor symptoms (bulbar, upper limb, lower limb, respiratory muscles, or multiple regions), main cognitive symptoms (behavioral abnormalities, language impairment, executive dysfunction, memory impairment), FTLD subtype or the frequency of HS.

**Table 1:** Comparison of clinical and pathological features between the amyotrophic lateral sclerosis (ALS) cases with or without comorbid pathology

	Total *N* = 29	Comorbid pathology (-) *N* = 12	Comorbid pathology (+) *N* = 17	*P* value
Age at onset (years)	67.5 (60.75–70.5)	66 (55–58)	69 (67–72)	0.056
Age at death (years)	74 (68–79)	68 (58–76)	76 (71–79)	0.048
Sex (male)	19 (65)	7 (58.3)	12 (70.6)	0.694
Total disease duration (months)	40 (21.5–108.75)	39 (22.5–87.5)	47 (20–133)	0.925
Onset of cognitive or behavioral abnormality				
before motor symptom	15 (51.7)	6 (50.0)	9 (52.9)	1.000
after motor symptom	5 (17.2)	2 (16.7)	3 (17.6)	1.000
simultaneous	5 (17.2)	1 (8.3)	4 (23.5)	0.370
NA	4 (13.8)	3 (25.0)	1 (5.9)	0.279
Initial motor symptom				
bulbar	14 (48.3)	5 (41.7)	9 (52.9)	0.710
upper	6 (20.7)	5 (41.7)	1 (5.9)	0.056
lower	4 (13.8)	1 (8.3)	3 (17.6)	0.622
respiratory	1 (3.4)	0	1 (5.9)	1.000
multiple domains	2 (6.9)	0	2 (11.8)	0.498
NA	2 (6.9)	1 (8.3)	1 (5.9)	1.000
Main cognitive or behavioral symptom				
behavioral abnormality	20 (69.0)	8 (66.7)	12 (70.6)	1.000
language dysfunction	3 (10.3)	0	3 (17.6)	0.246
executive dysfunction	2 (6.9)	2 (16.7)	0	0.163
memory impairment	11 (37.9)	5 (41.7)	7 (41.2)	1.000
ALS type2 pathology	23 (79.3)	12 (100)	11 (64.7)	0.028
FTLD subtype				
Type A	2 (6.9)	2 (16.7)	0	0.163
Type B	21 (72.4)	10 (83.3)	11 (64.7)	0.408
Type C	0	0	0	1.000
Hippocampal sclerosis	14 (48.3)	6 (50.0)	8 (47.1)	1.000
Comorbid pathology				
AD	9 (31.0)	-	9 (52.9)	-
AGD	4 (13.8)	-	4 (23.5)	-
PART (≧ Braak NFT stage III)	4 (13.8)	-	4 (23.5)	-

Values are presented as n (%) or median (IQR). Statistical analysis was performed using the Mann–Whitney U test for age at death and disease duration, and Fisher’s test for other variables

Table 2 shows the clinicopathological profiles of the single cases of the total cohort. All ALS type 1 cases exhibited comorbid pathology. Accordingly, the cases were classified into three groups based on the type of ALS and the presence of comorbid pathologies: ALS type 1 with comorbid pathology (Cases 1–6), ALS type 2 with comorbid pathology (Cases 7–17), and ALS type 2 without comorbid pathology (Cases 18–29). Behavioral abnormalities and memory impairment were present across all groups at a high frequency. In contrast, language impairment and executive dysfunction were observed only in cases with ALS type 2 pathology. HS was found in 14 out of the 23 ALS type 2 cases and was not observed in ALS type 1. Comorbid AD pathology was clinically suspected in three cases, with cognitive symptoms preceding the onset of motor symptoms. None of the patients had familial neurological disorders.

**Table 2:** Clinicopathological profiles of the single cases of the total cohort. ※PART is described as a comorbid pathology with Braak NFT stage Ⅲ or higher

Case	ALS subtype	Comorbid pathology	Age at death	Sex	Disease duration (months)	Main cognitive or behavioral symptoms				Stage of TDP-43 pathology	FTLD subtype	HS	NFT Braak stage	Aβ Thal Phase	senile plaque	NIA-AA AD likelihood	AGD stage	α-synuclein
						behavioral abnormality	language dysfunction	executive dysfunction	memory impairment									
1	Type 1	AD	85	F	324	+	-	-	+	1	-	-	Ⅴ	5	Frequent	High	-	-
2	Type 1	AD	79	M	12	+	-	-	-	3	-	-	Ⅲ	3	Moderate	Intermediate	-	-
3	Type 1	AD	79	M	20	-	-	-	+	2	-	-	Ⅲ	3	Sparse	Intermediate	-	Brainstem
4	Type 1	AGD	68	F	23	+	-	-	-	3	-	-	Ⅲ	1	Sparse	Not	2	-
5	Type 1	PART^※^	71	F	62	+	-	-	-	1	-	-	Ⅲ	-	None	Not	-	-
6	Type 1	PART^※^	78	F	140	+	-	-	-	3	-	-	Ⅲ	-	None	Not	-	-
7	Type 2	AD	89	F	239	+	-	-	-	4	Type B	-	Ⅵ	5	Moderate	High	-	-
8	Type 2	AD	77	M	47	+	+	-	+	4	Type B	+	Ⅵ	5	Moderate	High	-	-
9	Type 2	AD	75	M	60	-	-	-	+	4	Type B	+	Ⅴ	5	Frequent	High	-	-
10	Type 2	AD	85	M	6	-	+	-	-	4	Type B	+	Ⅲ	3	Moderate	Intermediate	-	-
11	Type 2	AD	74	M	23	+	-	-	-	5	Type B	+	Ⅲ	4	Sparse	Intermediate	-	-
12	Type 2	AD	71	M	36	+	-	-	-	5	Type B	+	Ⅲ	5	Frequent	Intermediate	-	-
13	Type 2	AGD	76	M	4	-	-	-	+	4	Type B	+	Ⅲ	-	None	Not	3	-
14	Type 2	AGD	80	M	137	+	-	-	-	4	Type B	+	Ⅱ	-	None	Not	1	-
15	Type 2	AGD	65	M	69	+	+	-	-	4	Type B	-	Ⅰ	2	Sparse	Low	1	-
16	Type 2	PART^※^	70	F	133	-	-	-	+	4	Type B	+	Ⅲ	-	None	Not	-	-
17	Type 2	PART^※^	69	M	10	+	-	-	-	5	Type B	-	Ⅲ	1	None	Low	-	-

Case	ALS subtype	Comorbid pathology	Age at death	Sex	Disease duration (months)	Main cognitive or behavioral symptoms				Stage of TDP-43 pathology	FTLD subtype	HS	NFT Braak stage	Aβ Thal Phase	senile plaque	NIA-AA AD likelihood	AGD stage	α-synuclein
						behavioral abnormality	language dysfunction	executive dysfunction	memory impairment									
18	Type 2	-	45	M	11	-	-	+	+	4	Type B	-	Ⅰ	-	None	Not	-	-
19	Type 2	-	88	F	108	+	-	-	-	4	Type B	+	Ⅰ	2	Moderate	Low	-	-
20	Type 2	-	68	M	23	+	-	-	-	4	Type B	-	Ⅰ	-	None	Not	-	-
21	Type 2	-	68	F	19	+	-	-	-	4	Type B	-	Ⅰ	2	None	Not	-	-
22	Type 2	-	79	M	111	-	-	-	+	4	Type B	+	Ⅱ	-	None	Not	-	-
23	Type 2	-	58	F	22	-	-	+	+	4	Type B	+	Ⅱ	3	Moderate	Low	-	-
24	Type 2	-	70	F	39	+	-	-	-	4	Type B	-	Ⅰ	-	None	Not	-	-
25	Type 2	-	75	F	NA	+	-	-	-	4	Type B	+	Ⅰ	-	None	Not	-	-
26	Type 2	-	83	M	156	+	-	-	+	4	Type A	-	Ⅱ	-	None	Not	-	-
27	Type 2	-	66	M	67	+	-	-	-	4	Type A	+	Ⅱ	-	None	Not	-	-
28	Type 2	-	58	M	29	-	-	-	+	5	Type B	+	Ⅰ	-	None	Not	-	-
29	Type 2	-	45	M	41	+	-	-	-	5	Type B	-	Ⅰ	1	None	Not	-	-

## Discussion

Representative cortical involvement in ALS includes neuronal loss, astrogliosis, and superficial spongiosis [23]. Cognitive and behavioral abnormalities are significantly correlated with cortical TDP-43 pathology [4, 5, 24, 25]. In our cohort, 23 cases (79.3%) were classified as ALS type 2, with or without comorbid pathology. This finding suggests that most cognitive and behavioral abnormalities in ALS can be attributed to cortical involvement, which is consistent with previous reports [4, 5, 24, 25]. In contrast, six cases (20.7%) exhibited significant cognitive or behavioral abnormalities despite the absence of cortical TDP-43 pathology. This unexpected finding implies that additional pathological processes may underlie these symptoms. The overlap of neurodegenerative diseases is considered an important contributor to the heterogeneity of clinical symptoms [26]. Previous studies have reported that ALS is occasionally accompanied by additional neuropathological conditions, such as AD or LBD, which may complicate its clinical manifestations [6, 27]. Hence, the presence of additional neuropathological findings, particularly in ALS type 1 cases, may reasonably be considered to contribute to cognitive or behavioral abnormalities in ALS. In this study, comorbid AD, AGD, and PART were identified in all six ALS cases without cortical involvement, suggesting their potential contribution to the observed cognitive and behavioral abnormalities. 

AD may be the most notable neuropathological contributor to cognitive or behavioral abnormalities in ALS [6, 7]. Previous reports have described cases of high-likelihood AD pathology in the absence of cortical ALS involvement [6]. AD is the most common form of dementia and typically presents with memory impairment, although atypical clinical variants have also been described [28]. In our case series, AD-related pathology was observed in nine cases (Cases 1–3 and 7–12). Memory impairment was observed in three out of the four high-likelihood AD pathology cases, suggesting that AD pathology contributed to the observed symptoms. In addition, behavioral abnormalities with or without memory impairment were observed in approximately two-thirds of the cases with AD pathology. Behavioral abnormalities may appear to differ from the typical clinical presentation of AD; however, AD can also manifest with behavioral and psychological symptoms, which are described separately from cognitive decline and are referred to as behavioral and psychological symptoms of dementia (BPSD) [29]. The causes of BPSD are multifactorial, including environmental and physical factors. Notably, AD pathology has been linked to the emergence of BPSD even in the early stages of disease [30, 31]. Furthermore, in addition to physical disability, psychosocial stress and pain are common clinical issues in patients with ALS [32, 33]. These clinical factors increase the risk of BPSD [34]. In these present ALS cases showing behavioral abnormalities with comorbid AD pathology, the observed symptoms may have been driven by the AD pathology, a possibility that may be underrecognized and may complicate the clinical interpretation of behavioral abnormalities in ALS.

We found four (13.4%) cases with comorbid AGD pathology. ALS is sometimes accompanied by AGD [22]. And its contribution to the clinical manifestation of ALS remains unclear because AGD can be present in older adults with preserved cognitive function [35]. However, AGD was first described as non-AD dementia, and cognitive symptoms appear to be associated with AGD, especially in the advanced stage [22, 36]. In our series, only one case demonstrated AGD stage 3, and this case presented with memory impairment, which was consistent with the underlying pathology (Case 13). AGD has been associated with psychiatric symptoms [36–38], frequently with irritability [36]; bipolar disorder or late-onset schizophrenia [37, 38]; and psychiatric and cognitive symptoms, even at stage 2 or lower [37, 39]. Moreover, AGD is a risk factor for suicide regardless of the stage [40]. The amygdala is affected in the early stages of AGD, and the characteristic morphological features in this region include argyrophilic grains, ballooned neurons, granular fuzzy astrocytes, neuronal loss, and astrogliosis (Fig. 2G–I) [22]. The occurrence of psychotic symptoms in AGD cases may be associated with the initial involvement of the limbic system, such as the amygdala [37, 40]. Therefore, the development of behavioral abnormalities in the ALS type 1 cases with AGD stage 2 can be attributed to the underlying AGD pathology (Case 4).

PART is a pathological concept that describes limbic-predominant tau pathology in the absence of amyloid plaques, representing a continuum from cognitively normal aged individuals to those with dementia and encompasses SD-NFT [19]. Since PART is commonly identified at autopsy among elderly individuals without dementia, its contribution to cognitive symptoms should be interpreted carefully. SD-NFT is a form of dementia characterized by abundant NFTs in the limbic regions without amyloid-β deposition [18, 41]. Most patients diagnosed with SD-NFT are classified as NFT Braak stages III and IV [42, 43]. When comorbid pathology of PART NFT Braak stage III and IV is observed in autopsy cases of patients with dementia, its potential association with clinical dementia symptoms needs to be carefully considered as SD-NFT. The clinical presentation of SD-NFT resembles that of AD, and many cases are likely to be clinically misdiagnosed as AD unless confirmed by autopsy [43]. The progression of SD-NFT is generally slower than that of AD, and behavioral abnormalities emerge in the later stages of the disease [18, 41]. However, in psychiatric hospital cohorts, psychiatric symptoms, such as delusions, are common in SD-NFT, and the clinical manifestations observed in such cases may reflect the characteristics of the underlying cohort [42]. In our study, two ALS type 1 cases with PART, both classified as NFT Braak stage III, presented with behavioral abnormalities (Cases 5 and 6). A direct causal relationship cannot be established due to the mild degree of PART pathology and behavioral abnormalities are not specific symptom of SD-NFT. However, the presence of PART pathology in ALS type 1 cases with behavioral abnormalities suggests that its role in ALS should not be underestimated.

Memory impairment is not considered a specific cognitive symptom of ALS [9]. Initially, we hypothesized that memory impairment is significantly associated with comorbid pathologies. However, our results did not support this hypothesis, as approximately 40% of ALS type 2 cases without comorbid pathology exhibited memory impairment. Memory impairment is occasionally observed in patients with ALS and has been linked to hippocampal atrophy on neuroimaging [44, 45]. Advanced degeneration of the hippocampal perforant pathway, including the entorhinal region and subiculum in ALS, is associated with memory disturbances and mimics AD [13]. In this study, three of the five ALS type 2 cases without comorbid pathology or cases with memory impairment showed prominent HS (Cases 22, 23, and 28). These findings suggest that HS may contribute to memory impairment in ALS independent of comorbid pathologies. Thus, memory impairment did not reliably predict comorbid pathologies.

The coexistence of ALS with LBD is rare but occasionally observed [27]. Previous reports have described ALS cases with α-synuclein pathology presenting with parkinsonism or orthostatic hypotension, suggesting that the comorbidity of α-synuclein pathology may influence the clinical manifestations of ALS [27, 46]. However, in the present study, only one case exhibited a small number of α-synuclein-positive structures in the medulla oblongata; this case was regarded as an incidental finding or prodromal Parkinson’s disease, with little association with clinical symptoms.

This study has some limitations. Whether these comorbid pathologies contribute to developing cognitive or behavioral symptoms remains a matter of discussion. The prevalence of comorbid pathology was significantly higher among older individuals. AD, AGD, and PART pathologies have also been observed in cognitively unimpaired older individuals [47]. Therefore, these pathologies may represent age-related changes rather than pathological contributors in older patients with ALS. The sample size was small, and further classification by ALS type and comorbid pathology led to even smaller subgroups, precluding meaningful subgroup analyses. Due to the retrospective nature of this study, causal relationships could not be established. Detailed clinical information on ALS, such as the ALSFRS-R score, was not available. Cognitive symptoms were assessed based on clinical descriptions rather than standardized neuropsychological testing, which may have introduced variability in interpretation and reduced objectivity. In addition, selection bias related to autopsy consent may have been present. Despite these limitations, our autopsy-based findings provide important insights into the potential contribution of comorbid pathologies to ALS with cognitive and behavioral abnormalities. To determine the contribution of comorbid pathologies to cognitive and behavioral abnormalities in ALS, prospective studies with larger cohorts, standardized cognitive assessments, and comparisons with appropriate control groups are needed.

## Conclusion

A high frequency of comorbid pathologies is observed in elderly patients with ALS with cognitive or behavioral abnormalities. There are cases of ALS in which comorbid pathologies such as AD, AGD, and PART may contribute to cognitive or behavioral abnormalities, even in the absence of cortical TDP-43 pathology. HS of ALS may contribute to memory impairment independently of comorbid pathologies. These findings highlight the clinicopathological complexity of cognitive and behavioral manifestations in ALS and emphasize the need for comprehensive pathological evaluation. In the clinical practice of the ALS with cognitive or behavioral abnormalities, such symptoms are typically attributed to cortical TDP-43 pathology; however, the contribution of comorbid pathologies should not be underestimated.

## Data Availability

The datasets used or analysed during the current study are available from the corresponding author on reasonable request.

## Abbreviations

AD: Alzheimer’s disease
AGD: argyrophilic grain disease
ALS: amyotrophic lateral sclerosis
BPSD: behavioral and psychological symptoms of dementia
DLB: dementia with Lewy bodies
CERAD: consortium to establish a registry for Alzheimer’s disease
FTD: frontotemporal dementia
FTLD: frontotemporal lobar degeneration
HE: hematoxylin and eosin
HS: hippocampal sclerosis
IHC: Immunohistochemistry
LBD: Lewy body disease
NA: not available
NFT: neurofibrillary tangle
NIA-AA: National Institute on Aging-Alzheimer Association
PART: primary age-related tauopathy
pTau: phosphorylated tau
TDP-43: transactive response DNA-binding protein 43
